# Quantitative Analysis of PiB-PET with FreeSurfer ROIs

**DOI:** 10.1371/journal.pone.0073377

**Published:** 2013-11-06

**Authors:** Yi Su, Gina M. D'Angelo, Andrei G. Vlassenko, Gongfu Zhou, Abraham Z. Snyder, Daniel S. Marcus, Tyler M. Blazey, Jon J. Christensen, Shivangi Vora, John C. Morris, Mark A. Mintun, Tammie L. S. Benzinger

**Affiliations:** 1 Department of Radiology, Washington University School of Medicine, Saint Louis, Missouri, United States of America; 2 Division of Biostatistics, Washington University School of Medicine, Saint Louis, Missouri, United States of America; 3 Department of Neurology, Washington University School of Medicine, Saint Louis, Missouri, United States of America; 4 Knight Alzheimer's Disease Research Center, Washington University School of Medicine, Saint Louis, Missouri, United States of America; 5 Avid Radiophamaceuticals, Philadelphia, Pennsylvania, United States of America; Banner Alzheimer's Institute, United States of America

## Abstract

*In vivo* quantification of β-amyloid deposition using positron emission tomography is emerging as an important procedure for the early diagnosis of the Alzheimer's disease and is likely to play an important role in upcoming clinical trials of disease modifying agents. However, many groups use manually defined regions, which are non-standard across imaging centers. Analyses often are limited to a handful of regions because of the labor-intensive nature of manual region drawing. In this study, we developed an automatic image quantification protocol based on FreeSurfer, an automated whole brain segmentation tool, for quantitative analysis of amyloid images. Standard manual tracing and FreeSurfer-based analyses were performed in 77 participants including 67 cognitively normal individuals and 10 individuals with early Alzheimer's disease. The manual and FreeSurfer approaches yielded nearly identical estimates of amyloid burden (intraclass correlation = 0.98) as assessed by the mean cortical binding potential. An MRI test-retest study demonstrated excellent reliability of FreeSurfer based regional amyloid burden measurements. The FreeSurfer-based analysis also revealed that the majority of cerebral cortical regions accumulate amyloid in parallel, with slope of accumulation being the primary difference between regions.

## Introduction

The prevalence of Alzheimer's disease (AD) is expected to increase dramatically worldwide within the next 50 years [1]. The future success of disease-modifying therapies will depend on accurate early diagnosis before the onset of clinical symptoms [2], [3], [4]. Amyloid-beta (Aβ) plaque deposition is a hallmark of AD [5], [6]. With the development of positron emission tomography (PET) tracers with high affinity for Aβ plaques, such as ^11^C-Pittsburgh Compound B (PiB), it is now possible to quantify neuropathology that was previously detectable only by post-mortem examination [7]. PET enables *in vivo* visualization of AD pathology and allows for a broad range of metabolic processes to be assessed in preclinical and clinical AD. Individuals with AD and mild cognitive impairment have been shown to have elevated PiB retention in the cerebral cortex [7], [8], [9] although elevated PiB retention is also observed in some cognitively normal individuals. Aβ deposition in asymptomatic individuals may represent a preclinical biomarker of AD [10], [11]. Therefore, it is critical to quantify the Aβ burden accurately and robustly, to further our understanding of disease mechanisms and to develop early diagnostic techniques.

Various imaging protocols and analysis procedures currently exist for PiB PET imaging. Our approach utilizes a 60-minute dynamic PiB scan. Binding potentials (BP_ND_) are calculated using Logan graphical analysis [12] with cerebellar cortex as the reference region [4], [11], [13]. Manually defined regions of interest (ROIs) routinely examined include: prefrontal cortex (PREF), lateral temporal cortex (TEMP), precuneus (PREC), occipital lobe (OCC), head of the caudate (CAU), gyrus rectus (GR), cerebellum (CER), and brainstem (BS), with a predetermined set of rules for ROI delineation using co-registered MR images [11]. Based on these ROIs, our laboratory defines the mean cortical binding potential (MCBP) value as the mean BP_ND_ in PREF, PREC, TEMP, and GR [11]. Other investigators may use a dynamic scan of 90 minutes with a distribution volume ratio (DVR) value calculated using cerebellum as the reference region [14], [15], [16] and a different selection of manually defined ROIs. Additional technical variations include, but are not limited to, the use of standard uptake value ratio [17], [18], [19] and voxel-wise analyses [20], [21]. Due to the variation in imaging and data analysis protocols, it is not known whether findings from different research groups can be meaningfully compared. One key difficulty in achieving a standard protocol is dependence on manually drawn regions. One laboratory has reported good inter-rater reliability (in 5 control and 5 AD individuals) [22], but reproducibility was limited to the same research group. It should also be noted that, in many amyloid imaging studies using either hand drawn regions [11], [13], [14], [15], [17], [19] or automatic templates [22], [23], [24], the rationale for ROI selection typically has been based on which regions have been previously reported as selectively affected by AD [11], [16], [24]. Other regions have generally been overlooked except in voxel-based analysis [21], [25].

This study has three aims. First, we develop an automated, regional, quantitative amyloid imaging analysis protocol using FreeSurfer (Martinos Center for Biomedical Imaging, Charlestown, Massachusetts). We demonstrate that this protocol generates global amyloid deposition measurements comparable to results obtained with conventional hand drawn regions. FreeSurfer automatically segments and parcellates T1-weighted brain MRIs [26], [27], [28]. This tool has been used in many neuroimaging studies, including those focused on AD [29], [30], [31]. As a second aim, we examine test-retest reliability of the FreeSurfer based technique by analyzing the same PiB scan with FreeSurfer segmentation results from two consecutive MR scans. Finally, we investigate the distribution of amyloid deposition in FreeSurfer-defined cortical, subcortical and white matter regions of interest throughout the brain. Since the start of this work, a few groups has published their research using FreeSurfer to facilitate PiB imaging quantification [32], [33], [34], the relationship of FreeSurfer based quantification to manual based quantification has not been examined. It is also unknown how much the uncertainty in FreeSurfer segmentation would affect PiB quantification. We examine both of these two questions in this study.

## Methods

### I. Participants

Seventy-seven individuals (G1) aged 48 to 90 years old were selected from a larger population enrolled at the Washington University Knight Alzheimer's Disease Research Center (KADRC) in longitudinal studies of memory and aging. This cohort comprised 49 females and 28 males; 27 individuals were APOE4+ and 50 individuals were APOE4-. G1 includes representative participants across age and PiB status. Individuals were not excluded based on imaging findings; one individual had encephalomalacia, which provided a useful comparison between the manual and automated approaches. The clinical assessment protocol has been previously described [11], [35], [36]. In brief, a clinician determines the presence or absence of dementia and rates the severity in accordance with the Clinical Dementia Rating (CDR). CDR 0 indicates no cognitive impairment and CDR 0.5, 1, 2 and 3 indicate very mild, mild, moderate and severe dementia [35]. Our study included 67 non-demented individuals (CDR 0) and 10 individuals with very mild or mild dementia of the Alzheimer's type (CDR 0.5 or 1). All imaging was performed between 2005 and 2010.

A separate group of forty individuals (G2) aged 46 to 79 years old were selected from our KADRC participants for an MRI test-retest study. This cohort consisted of 29 females and 11 males; 15 individuals were APOE4+ and 24 were APOE4-; three individuals had CDR rating of 0.5; one individual had no APOE status or CDR rating.

#### I.1 Ethics statement

All assessment and imaging procedures were approved by Washington University's Human Studies Committee. Written informed consent was obtained from all individuals or their care givers.

### II. Imaging

In both cohorts, human brain PET imaging for amyloid deposition was performed using the radiotracer N-methyl-[^11^C]2-(4-methylaminophenyl)-6-hydroxybenzothiazole (PiB). Preparation of PiB was carried out according to the published protocol [37]. Dynamic PET imaging was conducted with a Siemens 962 HR+ ECAT scanner in three-dimensional mode after intravenous administration of approximately 12mCi of PiB. The images were reconstructed on a 128×128×63 matrix (2.12×2.12×2.43 mm) using filtered back-projection. Typical dynamic scans had 25×5 seconds frames, 9×20 seconds frames, 10×1 minute frames, and 9×5 minutes frames.

For G1, anatomic MRI images were acquired with T1-weighted magnetization-prepared rapid gradient echo (MPRAGE) sequence (1 mm isotropic voxels) variably using a Siemens Trio 3T scanner (N = 72), a Siemens Vision 1.5T (N = 3), or a Siemens Avanto 1.5 T scanner (N = 2). For G2, two MPRAGE scans were acquired during the same MR session for each participant on the Siemens Trio 3T scanner to investigate the impact of FreeSurfer segmentation variability on PET quantification.

### III. Manual ROI analysis

**Figure 1:**
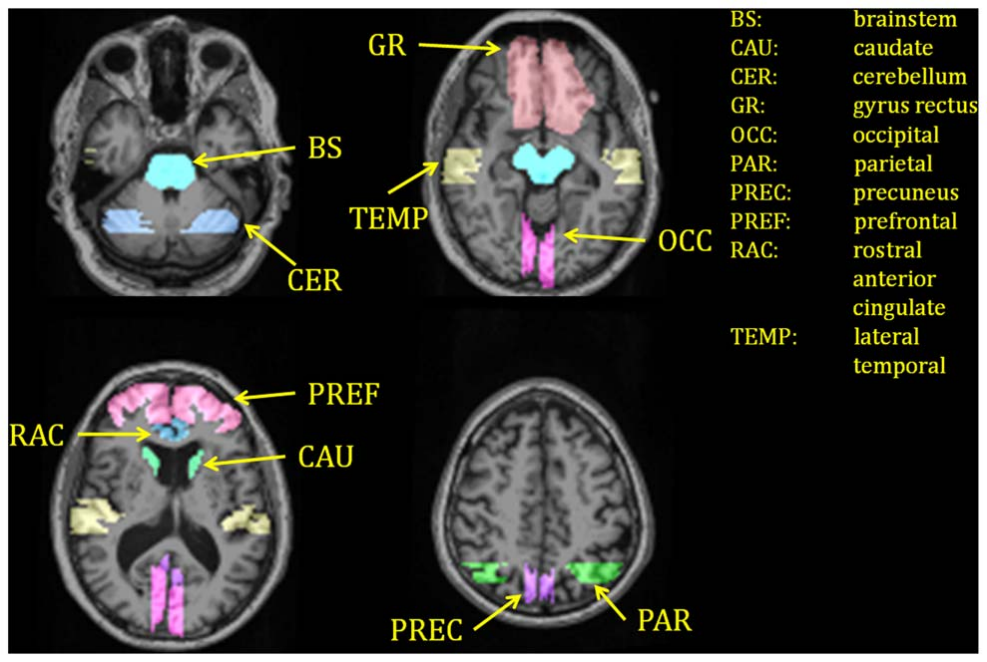
Example of regions-of-interest (ROI) defined manually on one of individual.

ROIs (Fig. 1) were manually defined according to previously described rules [11] using ANALYZE^TM^ software [38] and MRI images previously transformed (12-parameter affine) to atlas space [39]. These regions were originally selected through review of the 30 to 60 minute PiB PET images in Alzheimer individuals to optimize the detection of elevated PiB uptake [11]. PET-MR registration was performed using the VGM algorithm [40]. Manually defined ROIs were then transformed to the native PET space. Inter-frame motion correction for the dynamic PET images was performed using standard image registration techniques [41] implemented in in-house software [39]. Regional time-activity curves for each ROI were extracted by resampling the ROIs on the co-registered, unblurred PET images. Regional binding potentials (BP_ND_) were estimated using Logan graphical analysis [12] with cerebellar cortex as reference [42]. The average of BP_ND_ from four regions (PREF, PREC, GR, and TEMP) determined the mean cortical binding potential (MCBP) [11]. The washout rate constant (k_2_) of the reference region (cerebellum) was set to 0.16/minute. It has previously been shown that varying k_2_ over a 10-fold range (0.05 to 0.5/minute) has minimal impact on the BP_ND_ values [11].

### IV. FreeSurfer based analysis

FreeSurfer 5.0 was used to automatically segment the brain into various regions for G1 (as defined in the wmparc.mgz file); FreeSurfer 5.1 was used for brain segmentation for G2. Visual inspection of the automated segmentation results was performed for quality assurance purposes in all datasets. Correction was done when necessary according to the FreeSurfer manual (http://surfer.nmr.mgh.harvard.edu/fswiki/). Corresponding regions from the left and right hemispheres of the brain were combined to form a single ROI, e.g., the Left-Cerebellum-Cortex and the Right-Cerebellum-Cortex were combined to form a single ROI for quantitative analysis. The procedures used to compute BP_ND_ values from FreeSurfer-defined and manually traced ROIs were otherwise identical.

To generate a comparable global amyloid deposition index similar to MCBP from our manual region approach, volumetric analysis was performed to identify FreeSurfer cortical regions maximally overlapping the manual ROIs (Table 1). To estimate the FreeSurfer version of MCBP (MCBP_FS), the FreeSurfer counterparts of the four manual regions (PREC_FS, PREF_FS, GR_FS, TEMP_FS) for MCBP calculation were used in the same fashion as in the manual technique.

**Table 1:** Manual ROIs and their FreeSurfer counterpart.

Manual ROI	FreeSurfer ROI	Dice Coefficient	
CAU	Caudate	0.32	
CER	Cerebellum-Cortex	0.15	
GR	ctx-lateralorbitofrontal	0.14	0.20 (combined)
	ctx-medialorbitofrontal	0.15	
OCC	ctx-lingual	0.10	0.16 (combined)
	ctx-cuneus	0.10	
PAR	ctx-inferiorparietal	0.09	
PREC	ctx-precuneus	0.18	
PREF	ctx-rostralmiddlefrontal	0.13	0.13 (combined)
	ctx-superiorfrontal	0.07	
RAC	ctx-rostralanteriorcingulate	0.21	
TEMP	ctx-superiortemporal	0.13	0.18 (combined)
	ctx-middletemporal	0.11	

CAU = caudate; CER = cerebellum; GR = gyrus rectus; OCC = occipital cortex; PAR = parietal cortex; PREC = precuneus; PREF = prefrontal cortex; RAC = rostral anterior cingulate; TEMP = lateral temporal cortex. FreeSurfer ROI lists the combined FreeSurfer left and right region.

### V. Partial volume correction

In addition to analysis based on raw regional time-activity curves, partial volume corrected results were also obtained for G1 using a two-component technique [43] that has been widely applied in the context of PiB data analysis [14], [16], [17]. A brain tissue mask is generated based on FreeSurfer segmentation, a CSF dilution factor is calculated for each region, and the raw time-activity curve for each region is corrected by this dilution factor before BP_ND_ is calculated.

### VI. Test-retest study (G2)

For G2, we processed the same PiB dataset with FreeSurfer ROIs generated based on the two different MPRAGE scans. A mean test-retest variability measurement ΔBP% was calculated for each region and MCBP according to Eq. 1:
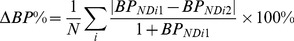
(1)where, N is the total number of participants (40), *i* is the index for individual patients, BP_NDi1_ and BP_NDi2_ are the estimated BP_ND_ using the first and second MPRAGE, respectively. In addition, a volumetric variability measurement Δ*VOL*% was also calculated for each region based on the repeated MPRAGE and FreeSurfer outputs following Eq. 2:
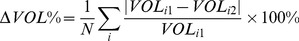
(2)where, *VOL_i1_* and *VOL_i2_* are the total number of voxels in each FreeSurfer region obtained with the first and second MPRAGE.

### VII. Statistical analysis

Intra-class correlation coefficients (ICC) were calculated to examine the agreement between binding potentials estimated using the manual and FreeSurfer approaches. SAS software (SAS Institute Inc., Cary, North Carolina, USA) was used to calculate the ICC estimates and their confidence intervals. We adjusted for CDR status (CDR = 0: negative, CDR>0: positive), age, and ApoE4 status. ApoE4 status was defined as 0 (no copies ApoE4) or 1 (at least 1 copy of the ApoE4 allele). To adjust for these covariates, mixed models with a variance components structure were employed to estimate the ICC and 95% confidence intervals. We specified a random intercept to account for the within-subject correlation caused by each subject having two regional binding potential observations. In addition, we treated rater as a random effect. The variance components estimated from the mixed model provided an ICC estimate. ICC was estimated as 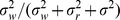, where 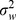 is the within-subject variance, 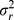 is the within-rater variance, and 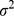 is the residual variance.

In the MRI test-retest study, in addition to test-retest variability as defined by Eqs. 1 and 2, ICC was also calculated for repeated measurements of BP_ND_ and FreeSurfer regional volumes for comparison with previously reported results.

To examine regional amyloid binding patterns, the Pearson correlation coefficient was evaluated across subjects between the regional BP_ND_ and the MCBP. Pearson correlation was also evaluated between cortical gray matter regions and the underlying white matter regions. Both Pearson correlation and Spearman correlation were evaluated for BP_ND_ estimated with and without partial volume correction.

We have previously used a manual MCBP cutoff of 0.18 as the criterion for PiB status determination [11], [44], [45]. To investigate the impact of using the FreeSurfer-based PiB quantification technique to assess PiB status, we also examined the feasibility of classifying participants as either PiB- or PiB+ using FreeSurfer-based global or regional binding potentials. These classifications were compared with results obtained by the manual MCBP approach.

### VIII. Software

The FreeSurfer-based analysis workflow has been implemented as an open source package that can be run from a linux command line or through the XNAT imaging informatics platform [46]. Specific modules include PET quantification and partial volume correction (C source code), a toolbox for image registration and analysis (C and Fortran), and a Unix shell script for executing the full workflow. The source code for the partial volume correction is available at (https://bitbucket.org/nrg/fs_tools). The XNAT module includes: a pipeline for executing the workflow, data types for representing the FreeSurfer and MCBP output, and web-based reports for displaying quality control and data reports. The XNAT module can be accessed on the XNAT Marketplace at https://marketplace.xnat.org/fspet.

## Results

### I. Manual vs. FreeSurfer region definitions

**Figure 2:**
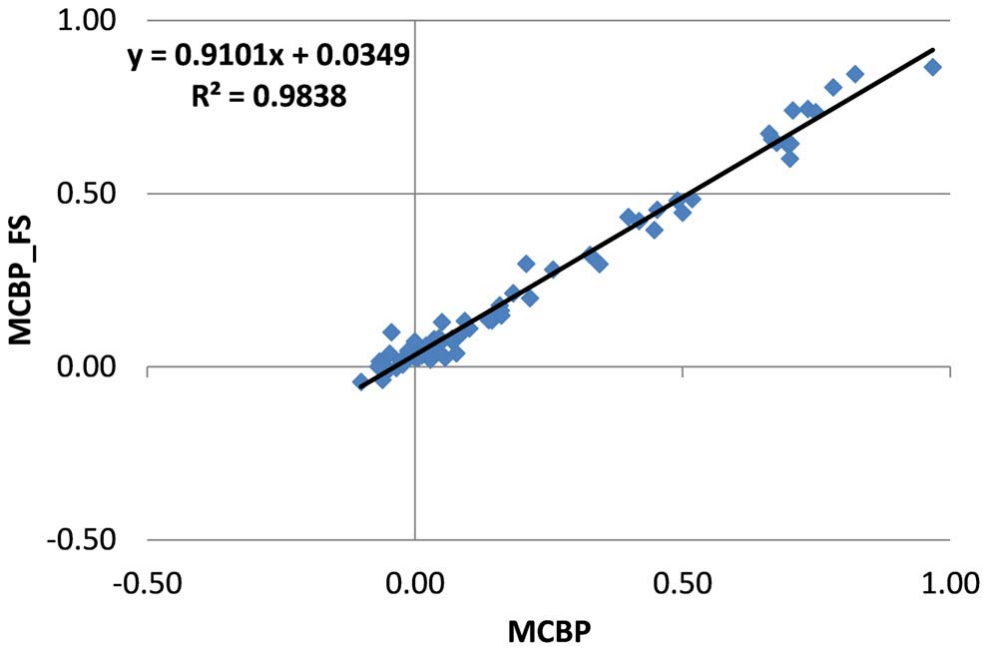
Scatter plot of MCBP values obtained using manually and FreeSurfer defined ROIs (previously shown in [47]).

Excellent agreement in MCBP measurement was observed between the manual and FreeSurfer based approaches without partial volume correction (ICC = 0.98 (95%CI: 0.97, 0.99)) (A recent review (in Russian) [47] briefly mentioned our technique and a modified version of Figure 2 was shown to demonstrate the FreeSurfer based quantification method as an effective approach for routine analysis of amyloid PET imaging data). The results obtained by both methods were highly correlated (Pearson r = 0.99, p<10^−68^, MCBP_FS = 0.91×MCBP_MAN+0.03; Spearman r = 0.94). These results were generated without considering the MR scanner differences. The same outcome was obtained controlling for variability in MR scanners and excluding the 5 subjects scanned at 1.5T. Therefore, all the analyses presented below were based on all the participants without controlling for MR scanner differences. When partial volume correction was applied, agreement was still excellent although ICC decreased slightly (ICC = 0.95, 95%CI: 0.94, 0.96). With partial volume correction, the Pearson correlation between the two approaches was 0.99 (p<10^−68^), and Spearman correlation was 0.92.

To categorize subjects as PiB- vs. PiB+, a MCBP cutoff value of 0.18 has been used in previous studies [11], [44], [45]. Using the same cutoff, the present cohort was separated into 52 PiB- subjects and 25 PiB+ subjects based on the conventional manual approach. Determination of PiB status was identical using FreeSurfer ROIs and the same 0.18 cutoff, which further demonstrates the equivalence of the two approaches.

### II. MRI Test-retest reproducibility

Test-retest data are listed in Table 2. FreeSurfer segmented ROI volumes varied by a few percent (nominally, ∼5%) on repeat MRI. ICC values for ROI volume ranged from 0.684 for the frontal pole to 0.996 for Unsegmented White Matter. However, BP_ND_ measurements were remarkably stable (ranged 0.25% for MCBP to 1.91% for CC_Mid_Posterior). Test-retest reproducibility of BP_ND_ assessed by ICC was excellent: the minimum ICC was 0.970 for CC_Central; in several regions, including MCBP and posterior cingulate cortex, test-retest ICC was 1.0.

**Table 2:** Test-retest reliability of FreeSurfer based PiB quantification in G2.

ROI	ΔBP%	ΔVOL%	ICC_BP (95% CI)	ICC_VOL (95% CI)
Cerebellum-White-Matter	0.71	3.88	0.978(0.958;0.988)	0.920(0.855;0.957)
Thalamus-Proper	0.68	3.06	0.990(0.981;0.995)	0.930(0.871;0.962)
Caudate	0.44	1.66	0.999(0.999;1.000)	0.987(0.975;0.993)
Putamen	0.26	2.38	1.000(0.999;1.000)	0.965(0.935;0.981)
Pallidum	0.86	4.91	0.981(0.965;0.990)	0.847(0.730;0.916)
Hippocampus	0.28	1.67	0.998(0.997;0.999)	0.983(0.968;0.991)
Amygdala	0.65	3.41	0.994(0.988;0.997)	0.959(0.920;0.979)
Accumbens-area	1.12	7.51	0.997(0.995;0.999)	0.854(0.741;0.920)
VentralDC	0.42	2.36	0.995(0.990;0.997)	0.931(0.873;0.963)
choroid-plexus	1.03	4.96	0.993(0.986;0.996)	0.959(0.923;0.978)
Brain-Stem	0.34	1.12	0.996(0.993;0.998)	0.989(0.980;0.994)
CC_Posterior	0.99	1.55	0.988(0.977;0.994)	0.994(0.988;0.997)
CC_Mid_Posterior	1.91	2.81	0.985(0.972;0.992)	0.987(0.976;0.993)
CC_Central	1.83	3.58	0.970(0.944;0.984)	0.954(0.916;0.976)
CC_Mid_Anterior	1.29	2.12	0.995(0.990;0.997)	0.986(0.974;0.993)
CC_Anterior	0.71	1.94	0.993(0.986;0.996)	0.991(0.984;0.995)
ctx-bankssts	0.63	4.59	0.999(0.998;0.999)	0.911(0.838;0.952)
ctx-caudalanteriorcingulate	0.43	3.49	0.999(0.999;1.000)	0.982(0.966;0.990)
ctx-caudalmiddlefrontal	0.53	3.46	0.999(0.999;1.000)	0.980(0.962;0.989)
ctx-cuneus	0.53	3.52	0.997(0.993;0.998)	0.919(0.853;0.956)
ctx-entorhinal	1.21	6.34	0.979(0.961;0.989)	0.927(0.866;0.960)
ctx-fusiform	0.40	2.54	0.999(0.998;0.999)	0.973(0.949;0.986)
ctx-inferiorparietal	0.51	2.21	0.999(0.999;1.000)	0.971(0.946;0.984)
ctx-inferiortemporal	0.54	2.54	0.999(0.998;1.000)	0.971(0.946;0.985)
ctx-isthmuscingulate	0.56	2.88	0.999(0.998;0.999)	0.951(0.910;0.974)
ctx-lateraloccipital	0.55	2.08	0.997(0.995;0.999)	0.973(0.949;0.985)
ctx-lateralorbitofrontal	0.45	2.43	0.999(0.999;1.000)	0.953(0.911;0.975)
ctx-lingual	0.37	1.78	0.998(0.996;0.999)	0.988(0.978;0.994)
ctx-medialorbitofrontal	0.69	3.13	0.999(0.998;1.000)	0.925(0.864;0.960)
ctx-middletemporal	0.47	2.26	0.999(0.999;1.000)	0.962(0.928;0.980)
ctx-parahippocampal	0.51	3.32	0.997(0.995;0.998)	0.938(0.887;0.967)
ctx-paracentral	0.46	3.64	0.999(0.998;1.000)	0.948(0.905;0.972)
ctx-parsopercularis	0.43	2.33	1.000(0.999;1.000)	0.982(0.966;0.990)
ctx-parsorbitalis	0.71	2.68	0.999(0.999;1.000)	0.954(0.915;0.975)
ctx-parstriangularis	0.56	3.07	0.999(0.999;1.000)	0.966(0.936;0.982)
ctx-pericalcarine	0.46	3.99	0.998(0.996;0.999)	0.941(0.891;0.968)
ctx-postcentral	0.45	3.53	0.999(0.998;0.999)	0.939(0.888;0.967)
ctx-posteriorcingulate	0.39	2.61	1.000(1.000;1.000)	0.970(0.945;0.984)
ctx-precentral	0.35	3.67	0.999(0.998;1.000)	0.910(0.837;0.952)
ctx-precuneus	0.31	2.11	0.999(0.999;1.000)	0.973(0.951;0.986)
ctx-rostralanteriorcingulate	0.53	3.50	1.000(0.999;1.000)	0.978(0.958;0.988)
ctx-rostralmiddlefrontal	0.45	2.09	1.000(0.999;1.000)	0.978(0.958;0.988)
ctx-superiorfrontal	0.34	2.00	1.000(1.000;1.000)	0.966(0.936;0.982)
ctx-superiorparietal	0.41	2.29	0.999(0.999;1.000)	0.973(0.949;0.985)
ctx-superiortemporal	0.33	1.50	1.000(0.999;1.000)	0.984(0.968;0.992)
ctx-supramarginal	0.33	2.54	1.000(0.999;1.000)	0.961(0.928;0.979)
ctx-frontalpole	1.65	8.76	0.995(0.990;0.997)	0.684(0.476;0.820)
ctx-temporalpole	1.45	6.03	0.988(0.978;0.994)	0.811(0.670;0.896)
ctx-transversetemporal	0.46	4.93	0.999(0.998;0.999)	0.920(0.854;0.957)
ctx-insula	0.32	3.01	1.000(0.999;1.000)	0.939(0.889;0.967)
UnsegmentedWhiteMatter	0.29	1.32	0.998(0.996;0.999)	0.996(0.993;0.998)
GR_FS	0.47	1.85	0.999(0.999;1.000)	0.970(0.944;0.984)
TEMP_FS	0.34	1.73	1.000(0.999;1.000)	0.975(0.951;0.987)
OCC_FS	0.37	1.92	0.998(0.996;0.999)	0.977(0.958;0.988)
PREF_FS	0.35	1.84	1.000(1.000;1.000)	0.976(0.955;0.987)
MCBP	0.25	1.27	1.000(1.000;1.000)	0.983(0.968;0.991)

### III. Partial volume correction

Partial volume corrected binding potential values strongly correlated with uncorrected values (Table 3). Thus, in most ROIs, partial volume correction did not cause major changes in subject ranking, as revealed by high values of Spearman correlation. Most rank changes occurred in subjects with low levels of PiB uptake. Lower Spearman correlations were observed in regions with low PiB retention and narrow ranges of BP_ND_ values (e.g., hippocampus).

**Table 3:** Correlations of binding potentials between raw measurements and partial volume corrected measurements for selected FreeSurfer regions and MCBP in G1.

Structure	slope	intercept	Pearson r	Spearman r
Cerebellum-White-Matter	0.544	0.012	0.798	0.747
Thalamus-Proper	0.862	−0.037	0.958	0.917
Caudate	0.985	0.037	0.980	0.934
Putamen	0.826	−0.134	0.989	0.958
Pallidum	0.744	−0.093	0.948	0.933
Hippocampus	0.506	−0.008	0.539	0.543
Amygdala	0.995	−0.051	0.872	0.875
Accumbens-area	0.953	−0.065	0.993	0.976
VentralDC	0.856	0.030	0.939	0.927
Brain-Stem	0.932	0.036	0.926	0.877
ctx-cuneus	0.925	0.176	0.878	0.800
ctx-inferiorparietal	1.187	0.142	0.993	0.929
ctx-lateralorbitofrontal	1.191	0.144	0.993	0.961
ctx-lingual	1.014	0.095	0.929	0.825
ctx-medialorbitofrontal	1.223	0.175	0.993	0.950
ctx-middletemporal	1.213	0.144	0.992	0.938
ctx-precuneus	1.138	0.089	0.990	0.940
ctx-rostralanteriorcingulate	1.124	0.133	0.981	0.881
ctx-rostralmiddlefrontal	1.240	0.219	0.994	0.944
ctx-superiorfrontal	1.218	0.183	0.992	0.915
ctx-superiortemporal	1.240	0.160	0.985	0.882
MCBP	1.202	0.142	0.995	0.955

Also listed were the slope and intercept of the linear fitting between raw measurements and partial volume corrected ones. All the correlations were statistically significant (p<10^−6^), correction for multiple comparison was not performed.

### IV. Regional specificity of PiB binding

**Figure 3:**
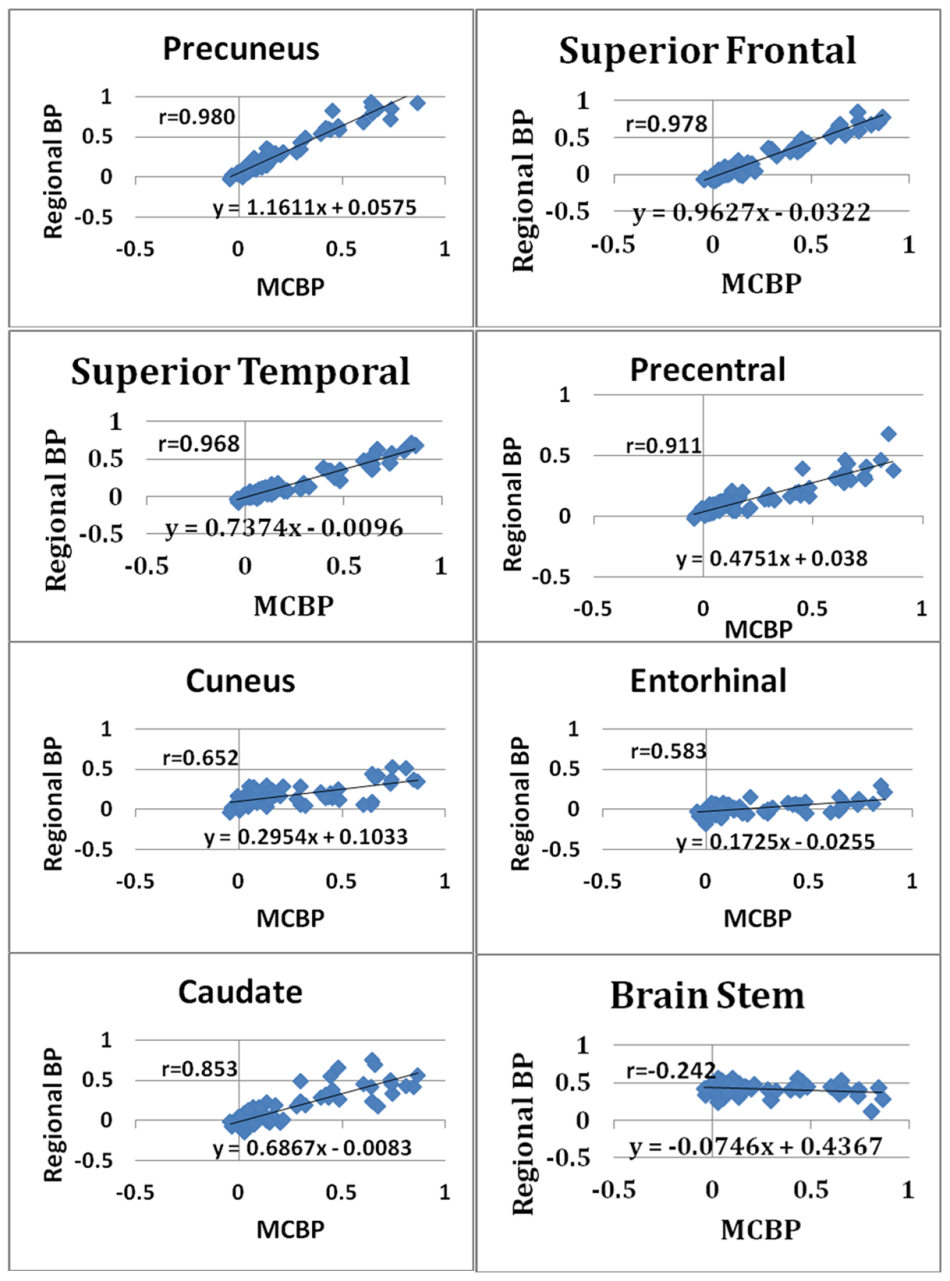
Correlation of regional binding potential and MCBP for selected regions.

**Figure 4:**
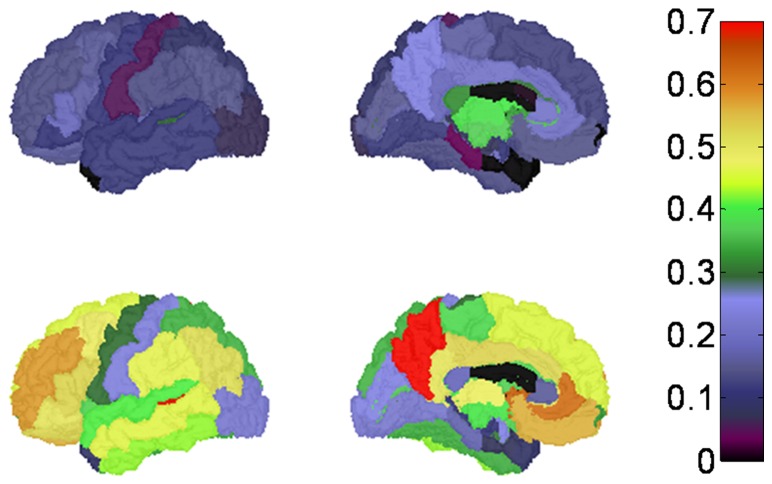
Average regional amyloid deposition for the CDR0 group (top row) and the CDR+ group (bottom row) quantified using FreeSurfer regions. For each group the lateral (left) and medial (right) surfaces of the left hemisphere were illustrated.

Traditionally, PiB status has been determined by evaluating MCBP, computed by averaging BP_ND_ over a fixed set of ROIs [11], [14], [48]. For this purpose, our group has used four ROIs (see Introduction) [10], [11]. However, it is unclear whether the determination of PiB status is sensitive to this particular choice. To investigate this question, we evaluated regional BP_ND_ values in relation to our measure of MCBP. In the majority of the cortical regions, BP_ND_ values strongly correlated with the global MCBP (Table 4, Fig. 3). As might be predicted, regions with high levels of PiB binding in the clinically positive group (e.g., precuneus, BP_ND_  = 0.738±0.286 (mean ± SD) and rostral anterior cingulate, BP_ND_ = 0.657±0.295) (Fig. 4) showed the greatest correlation with MCBP (Pearson r = 0.98 and 0.96, respectively). Similarly, subcortical structures with high levels of PiB binding in the clinically positive group were also strongly correlated with MCBP, e.g., caudate (r = 0.853), putamen (r = 0.862), and accumbens (r = 0.913) (Table 5). Conversely, regions with lower BP_ND_, e.g., the cuneus gyrus and the entorhinal cortex, more weakly correlated with MCBP (Fig. 3). These lower correlations may reflect a different trajectory of amyloid accumulation over time in high vs. low BP_ND_ regions.

**Table 4:** Correlations of cortical regions binding potentials to MCBP and to their corresponding white matter regions in G1.

Structure	MCBP			white matter
	r	slope	intercept	r
ctx-rostralmiddlefrontal	0.984	1.227	−0.061	0.966
ctx-medialorbitofrontal	0.980	1.077	−0.028	0.965
ctx-precuneus	0.980	1.161	0.058	0.937
ctx-superiorfrontal	0.978	0.963	−0.032	0.940
ctx-lateralorbitofrontal	0.978	0.897	0.018	0.932
ctx-caudalmiddlefrontal	0.976	0.897	0.011	0.872
ctx-parstriangularis	0.976	0.934	0.030	0.937
ctx-parsorbitalis	0.973	1.019	−0.054	0.975
ctx-superiortemporal	0.968	0.737	−0.010	0.951
ctx-parsopercularis	0.966	0.903	0.024	0.926
ctx-supramarginal	0.966	0.880	−0.005	0.924
ctx-middletemporal	0.961	0.862	−0.018	0.964
ctx-posteriorcingulate	0.960	0.969	0.046	0.701
ctx-inferiorparietal	0.960	0.946	−0.003	0.953
ctx-rostralanteriorcingulate	0.955	1.132	0.015	0.846
ctx-caudalanteriorcingulate	0.951	0.960	0.033	0.686
ctx-superiorparietal	0.944	0.753	−0.032	0.933
ctx-inferiortemporal	0.942	0.774	−0.004	0.935
ctx-insula	0.938	0.651	0.019	0.808
ctx-bankssts	0.935	0.986	0.141	0.905
ctx-postcentral	0.919	0.535	−0.037	0.946
ctx-fusiform	0.914	0.551	0.049	0.882
ctx-precentral	0.911	0.475	0.038	0.807
ctx-paracentral	0.892	0.644	0.058	0.803
ctx-isthmuscingulate	0.884	0.757	0.065	0.650
ctx-transversetemporal	0.866	0.561	0.085	0.908
ctx-parahippocampal	0.859	0.400	−0.031	0.853
ctx-temporalpole	0.856	0.373	−0.109	0.872
ctx-lateraloccipital	0.772	0.436	−0.002	0.911
ctx-lingual	0.749	0.305	0.068	0.902
ctx-pericalcarine	0.742	0.437	0.103	0.880
ctx-cuneus	0.652	0.295	0.103	0.866
ctx-entorhinal	0.583	0.173	−0.025	0.680

All the correlations were statistically significant (p<10^−6^), correction for multiple comparison was not performed.

**Table 5:** Correlation of binding potentials to MCBP for subcortical structures (G1).

Structure	r	p-value	slope	intercept
Cerebellum-White-Matter	−0.252	0.027	−0.082	0.377
Thalamus-Proper	0.512	0.000002	0.204	0.359
Caudate	0.853	<10^−6^	0.687	−0.008
Putamen	0.862	<10^−6^	0.634	0.253
Pallidum	0.396	0.0004	0.162	0.440
Hippocampus	0.152	0.186	0.036	0.074
Amygdala	0.466	0.00002	0.151	0.095
Accumbens-area	0.913	<10^−6^	0.924	0.014
Substancia-Nigra	0.144	0.211	0.093	0.072
VentralDC	−0.054	0.639	−0.019	0.377
choroid-plexus	−0.189	0.100	−0.084	−0.193
Brain-Stem	−0.242	0.034	−0.075	0.437
CC_Posterior	−0.046	0.691	−0.031	0.310
CC_Mid_Posterior	−0.031	0.786	−0.024	−0.043
CC_Central	−0.088	0.448	−0.068	−0.037
CC_Mid_Anterior	−0.008	0.948	−0.005	−0.019
CC_Anterior	0.019	0.872	0.013	0.202

As noted earlier, previous studies have classified individuals as PiB- vs. PiB+ using MCBP >0.18 as the criterion [11], [44], [45]. We observed that many regions can be similarly used to classify individuals, provided an appropriate ROI-specific criterion is identified (Table 6). Among the FreeSurfer regions we examined, 26 cortical regions and 3 subcortical regions could be used to determine PiB positivity with less than 10% difference in classification using MCBP >0.18 as the reference. Identical classification was obtained based on the BP_ND_ in four regions, viz., ctx-medialorbitofrontal, ctx-parsorbitalis, ctx-rostralmiddlefrontal, and GR_FS.

**Table 6:** List of regions that have less than 10% difference in classifications for PiB positivity, and their corresponding BP_ND_ threshold, number of difference in classifications (NOD), and percentage difference in classification.

ROI	Threshold	NOD	D%
Caudate	0.173	4	5.19
Putamen	0.363	5	6.49
Accumbens-area	0.227	3	3.90
ctx-bankssts	0.373	6	7.79
ctx-caudalanteriorcingulate	0.265	4	5.19
ctx-caudalmiddlefrontal	0.170	2	2.60
ctx-inferiorparietal	0.170	5	6.49
ctx-inferiortemporal	0.159	7	9.09
ctx-isthmuscingulate	0.255	6	7.79
ctx-lateralorbitofrontal	0.177	2	2.60
ctx-medialorbitofrontal	0.224	0	0.00
ctx-middletemporal	0.137	4	5.19
ctx-paracentral	0.208	7	9.09
ctx-parsopercularis	0.172	3	3.90
ctx-parsorbitalis	0.146	0	0.00
ctx-parstriangularis	0.224	1	1.30
ctx-postcentral	0.050	7	9.09
ctx-posteriorcingulate	0.279	2	2.60
ctx-precentral	0.125	6	7.79
ctx-precuneus	0.303	3	3.90
ctx-rostralanteriorcingulate	0.278	2	2.60
ctx-rostralmiddlefrontal	0.174	0	0.00
ctx-superiorfrontal	0.171	3	3.90
ctx-superiorparietal	0.129	4	5.19
ctx-superiortemporal	0.145	6	7.79
ctx-supramarginal	0.142	4	5.19
ctx-frontalpole	0.023	2	2.60
ctx-transversetemporal	0.232	6	7.79
ctx-insula	0.133	4	5.19
GR_FS	0.202	0	0.00
TEMP_FS	0.136	4	5.19
PREF_FS	0.154	2	2.60

## Discussion

The main objective of this study was to examine the feasibility of using FreeSurfer-defined ROIs in place of manual regions for purposes of determining PiB status. A high level of agreement was found between the manual and FreeSurfer-based approaches to quantifying global amyloid burden using the MCBP. Moreoever, we observed high test-retest ICC for BP_ND_ measurements using FreeSurfer segmentations of repeated MRI scans. In fact, this ICC (>0.970) is better than the reported ICC values for inter-rater reliability and manual vs. automated comparison of regional PiB uptake measurements [22]. This indicates the FreeSurfer based PiB quantification is reliable in many regions and can therefore be routinely deployed. Some regions, e.g., the frontal pole, exhibit variable FreeSurfer volumes (test-retest ICC  = 0.684 in our data) [27]. Nevertheless, measured BP_ND_ was generally reliable, even in such regions (frontal pole ICC  = 0.995 in our data). It should be pointed out that the BP_ND_ test-retest reproducibility in this study only represents uncertainty attributable to region definition; we did not conduct a full test-retest study with repeated PiB scans as done by Lopresti and colleagues [16]. Uncertainty in BP_ND_ (<1% for most regions) attributable to FreeSurfer ROI definition variability is only a small fraction of the full test-retest variability reported by Lopresti et al. (∼5%) [16].

This study confirms the observation that amyloid deposition varies spatially [11], [15]. Traditionally, a small number of regions with the greatest PiB binding potentials have been used to evaluate PiB status. However, we find that many regions are comparably useful in determining PiB status, albeit with different thresholds (Table 6). This observation reflects the high correlation of regional BP_ND_ to MCBP in many regions (Table 4). The logic here is reminiscent of the demonstration by Haxby and colleagues that classification can be based on less robust features of imaging data [49], Thus, it is not critical to identify the “optimal” set of regions for determination of PiB status. Rather, we should focus on developing a standard approach to facilitate multi-institutional studies and cross comparisons of results from various groups.

It has not been standard practice in our group to apply partial volume correction in PiB studies. The two-component partial volume correction technique adopted by many groups [17], [22] compensates for the brain atrophy without modeling difference between gray vs. white matter. In a comparison study [50], it was demonstrated that three-component partial volume correction, which differentiates between gray vs. white matter, provides a more accurate estimation of regional intensity values. However, the three-component model was more sensitive to errors in image co-registration and segmentation. Therefore, it is not surprising that two-component partial volume correction did not change the rank of the amyloid burden measured by PiB PET, nor did it change correlation to MCBP within individual cortical regions. High correlations between cortical gray matter regions and the underlying white matter reflect the limited spatial resolution of PET. More sophisticated partial volume correction may enable detection of more localized variations in PiB retention. But these techniques must be thoroughly investigated to determine the impact of registration and segmentation errors.

## Conclusion

FreeSurfer-based ROI analysis has the advantage of automated segmentation, which greatly reduces labor costs and potentially enables standardization across laboratories. In addition, since FreeSurfer is widely used in AD research [29], [30], [51], a FreeSurfer-based amyloid imaging analysis protocol would allow integration of amyloid deposition measurements with cortical thickness, volume and other anatomical measurements. Although some degree of variability exists in the automated segmentation procedure [26], [28], [52], and manual correction of FreeSurfer-derived boundaries is sometimes necessary, especially in the presence of atrophy, our MRI test-retest study demonstrated excellent reliability of the FreeSurfer based estimation of regional BP_ND_ despite variability of ROI volumes. Our data also suggest that the majority of cerebral cortical regions accumulate amyloid in parallel. Longitudinal studies investigating the rate of amyloid accumulation both globally and regionally are ongoing in our laboratory.
